# Predicting productive, health, and reproductive traits in Mexican Holstein cattle using single nucleotide polymorphisms, haplotypes, and runs of homozygosity

**DOI:** 10.3168/jdsc.2025-0831

**Published:** 2025-12-13

**Authors:** José G. Cortes-Hernández, Guillermo Martinez-Boggio, Francisco Peñagaricano, Hugo H. Montaldo, Felipe J. Ruiz-López, Adriana García-Ruiz

**Affiliations:** 1 PhD Program in Animal Health and Production Science, National Autonomous University of Mexico, CDMX, 04510, Mexico; 2 Department of Animal Science, University of California, Davis, Davis, CA 95616; 3 Department of Animal and Dairy Sciences, University of Wisconsin–Madison, Madison, WI 53706; 4 Department of Genetics and Biostatistics, Faculty of Veterinary Medicine and Animal Husbandry, National Autonomous University of Mexico, CDMX, 04510, Mexico; 5 Faculty of Higher Studies Cuautitlán, National Autonomous University of Mexico, EDOMEX, 54714, Mexico; 6 National Center for Disciplinary Research in Animal Physiology and Improvement of the National Institute of Forestry, Agriculture and Livestock Research, Ajuchitlán, Querétaro, 76280, Mexico

## Abstract

The aim of this study was to evaluate the use of 3 different genomic relationship matrices built from SNPs, haplotypes (HAP), and runs of homozygosity (ROH), on phenotype predictive ability and estimated genetic variance of milk yield, SCS, and days open in Mexican Holstein cattle. The analyses included the use of the genomic relationship matrices as kernel-based models fitting either one or multiple sources of information. The SNPs and HAP matrices were built as linear kernels, and the ROH matrix as a Gaussian kernel. Also, we used as a reference the performance of the single-step GBLUP. Predictive ability was evaluated in 10-fold cross-validation. The highest predictive correlation was obtained using SNPs (0.63 for SCS, 0.57 for milk yield, and 0.20 for days open). The use of multigenomic relationships, including HAP and ROH, did not outperform the use of only SNPs in predictive ability, but the highest genetic variance was estimated using ROH (0.39 for milk yield, 0.26 for SCS, and 0.22 for days open).

## Highlights

• The use of ROH increases the estimation of genetic variance for health and reproductive traits.
• The combination of SNP, HAP, and ROH does not increase predictive performance.
• Kernel-based models (Bayesian) can capture greater genetic variance for complex traits.

## Graphical Abstract

**Figure:**
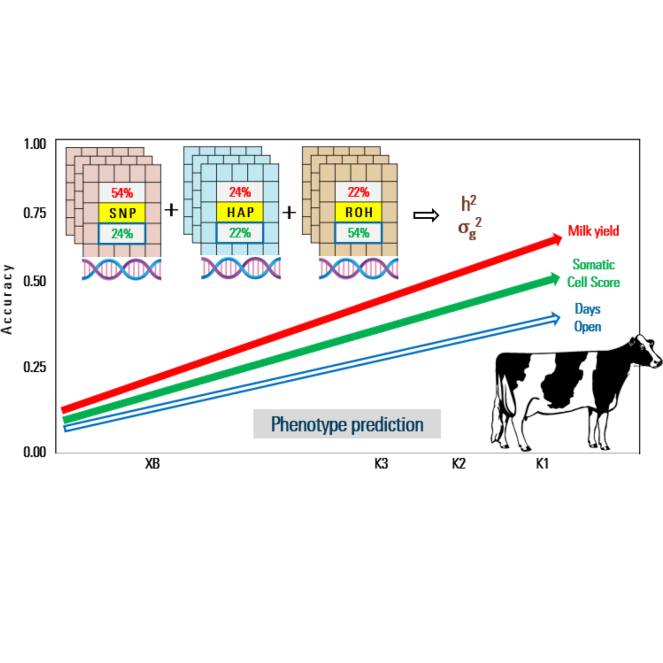
**Summary:** We investigated the use of different sources of genomic information, such as single nucleotide polymorphisms (SNPs), haplotypes (HAP), and runs of homozygosity (ROH), to predict productive, health, and reproductive traits in Mexican Holstein cattle. The use of models including genomic information from DNA markers increased the predictive performance and yielded higher estimates of genetic variance compared with models that included only fixed effects of complex traits in Mexican Holstein cattle.

High-throughput technologies have revolutionized dairy cattle genetics research, enabling the generation of multiple sources of genomic information, including SNPs, HAP, and ROH, as well as multi-omics data, such as genomics, transcriptomics, metabolomics, and proteomics. Their combination with phenotypic data such as production, health, and reproductive traits allows us to improve dairy cattle performance and informing breeding decisions (Zhu et al., 2022).

Health and reproductive traits are complex and often challenging to measure consistently on farms. Nonetheless, they are critical to the profitability of dairy farms. For example, SCS is a key indicator of udder health and milk quality, whereas days open is a measure of the interval between calving and subsequent conception, which directly affects reproductive efficiency, voluntary culling rates, and replacement costs (Temesgen et al., 2022; Ashja et al., 2024).

The use of multiple sources of genomic information is expected to increase the prediction accuracy of complex traits. These sources may capture various signals affecting phenotypes and thus could be combined to improve prediction performance. Previous research has shown the use of multiple sources of information, including SNP markers, HAP, and others omics data, increases the prediction of complex traits, such as fertility, milk composition, and feed efficiency (Baba et al., 2021; Martinez Boggio et al., 2024).

The use of an additive genetic relationship matrix based on SNPs is commonly used in genetic studies. Therefore, the integration of SNP, HAP, and ROH, individually (Ferdosi et al., 2016; Karimi et al., 2018) or combined, may represent an alternative strategy for enhancing phenotypic prediction, due to each genetic structure providing complementary information. For instance, HAP could identify genomic regions associated with specific functional traits and reveal genomic regions favored by natural or artificial selection that harbor genes relevant to the breed (Chen et al., 2018) and the use a genomic relationship matrix based on HAP, which could potentially capture interactions or information about genetic determinants with small effects that are not captured by individual SNPs (Teissier et al., 2020). The ROH are continuous segments of homozygous genotypes; these segments arise because the HAP were transmitted by a common ancestor sometimes influenced by genetic selection (Zhao et al., 2021) and can be used to build genomic matrices based on its presence or lack of presence. Likewise, ROH can be used to quantify the level of inbreeding and to infer aspects of a population's demographic and selection history, including past bottlenecks, inbreeding events, and selection pressures, based on their length and frequency across the genome (Zayas et al., 2024). Moreover, the amount of ROH segments increases the probability that deleterious recessive alleles become homozygous, potentially leading to reduced fertility, lower vigor, and greater disease susceptibility in cattle (Bhati et al., 2020). The ROH have been associated with productive, health, and reproductive traits in dairy cattle (Cortes-Hernández et al., 2022; Pacheco et al., 2023). Other studies in beef cattle have identified ROH islands on chromosomes 1, 3, 8, 11, 13, 14, and 16 where SNPs have been reported to be associated with growth, reproductive, milk production, residual feed intake, and meat quality traits (Zayas et al., 2024). Despite the use of ROH in association with economic traits, to the best of our knowledge, few studies have used them to perform phenotypic prediction in dairy cattle (Luan et al., 2014).

The objective of this study was to assess the phenotypic prediction of milk yield, SCS, and days open, by integrating SNPs, HAP, and ROH in Mexican Holstein cows. We hypothesized that the use of multiple sources of genomic information could enhance phenotypic prediction, especially when HAP and ROH capture variation that SNP data do not explain. We also evaluated the proportion of genetic variance explained by each of the sources of genomic information considered separately or in combination.

Data consisted of phenotypes and genotypes from 5,746 first-lactation Mexican Holstein cows. Cows were born between 2006 and 2016 from 271 herds. We evaluated 3 different phenotypic traits: milk yield as 305-ME (mature equivalent), SCS expressed as log_2_ (SCC/1,000,000) + 3, and days open defined as the number of days between calving and conception. Quality control consisted of including cows between 16 to 60 mo of age at first calving, and range of ±3 SD was established for milk yield, and a range of 30 to 400 d for days open. Only animals with all genomic and phenotype information were included. All cows had imputed genotype with 116,204 SNPs coming from different density commercial arrays (Illumina, Neogen, and Axiom): BovineLD v2.0 9K (4.15%), GGP Bovine LD v3.0 26K (5.91%), GGP Super LD v4.0 26K (1.38%), BovineSNP v3 50K (9.16%), GGP LD 77K (15.42%), GGP Bovine 100K (0.03%), GGP HD 150K (59.77%), Genome-Wide BOS 1 Bovine Array 640K (2.47%), and GGP HD 777K (1.71%), which were imputed using Findhap V2 (VanRaden et al., 2011; García-Ruiz et al., 2015).

From those 116,204 SNPs, the HAP were constructed following 3 steps: (1) we recoded SNPs as A/B (0 = BB; 1 = AB; 2 = AA; and 3, 4, and 5 = 00); (2) we defined HAP with at least 2 SNP per block and a value of linkage disequilibrium (**LD**; r^2^) ≥ 0.80 (Mucha et al., 2019); finally (3) each HAP was recoded as pseudoSNP 0/1/2 corresponding to the absence of paternal and maternal alleles, the presence of 1 copy or 2 copies, respectively. The included HAP had a minimum frequency of 1% in the population (Teissier et al., 2020). For detection, inference, and recodification of HAP as pseudoSNP we used PLINK 1.07 software (Purcell et al., 2007; Calderón-Chagoya et al., 2023). Although this version of PLINK may have limitations in HAP inference, we compared the haplotypes found with PLINK 1.9 (Chang et al., 2015) and obtained the same results. Another option is to use Beagle v5.1 software package for haplotype inference (Browning et al., 2018; Bian et al., 2021). After quality control to HAP and SNPs (call rate <0.95, minor allele frequency <0.05, and Hardy–Weinberg equilibrium test <0.15), we retained 88,911 SNPs and 35,552 HAP. Furthermore, we detected ROH using PLINK 1.9 software (Chang et al., 2015) with the following parameters: –bfile–cow–geno 0.1–mind 0.1–homozyg group–homozyg-gap 500–homozyg-window-het 1–homozyg-window-missing 1–homozyg-window-snp 50–not-chr X (Meyermans et al., 2020; Pacheco et al., 2023). We found a total of 111,084 ROH, of which 17.5% were present in more than one animal; the number of ROH per animal was 32.74 ± 6.94, with an average length and covered genome of 7.38 ± 6.85 Mb and 241.80 ± 75.13 Mb, respectively. Previous details of ROH in the Holstein population of Mexico were published by Cortes-Hernández et al. (2022). After ROH detection, each ROH was recorded as 0 or 1 according to the absence or presence in each animal, leaving a total of 106,082 ROH for milk yield, 102,427 for SCS, and 89,419 for days open, allowing at least one ROH per animal; the difference in the number of ROH is due to the different number of records by trait.

For prediction analysis, only animals with all genomic and phenotype information were included, records of 5,746 cows with milk yield, 5,508 of SCS, and 4,707 records of days open, and we computed 3 genomic relationship matrices like kernels including SNPs, HAP, and ROH data for each trait. The prediction models were defined as follows:

[1] $$y=\mathrm{HYS}\;+\;\mathrm{AGE}+\mathrm{AG}E^{2}+e,$$

[2] $$y=\mathrm{HYS}+\mathrm{AGE}+\mathrm{AG}E^{2}+K_{1}u+e,$$

[3] $$y=\mathrm{HYS}+\mathrm{AGE}+\mathrm{AG}E^{2}+K_{1}u+K_{2}u+e,$$

[4] $$y=\mathrm{HYS}+\mathrm{AGE}+\mathrm{AG}E^{2}+K_{1}u+K_{2}u+K_{3}u+e,$$

where y is the analyzed trait (MY, SCS, and days open), **HYS** is the vector of herd-year-season (including 113 levels for milk yield, 105 for SCS, 95 for days open, with 50.85 ± 31.35 animals on average, and a minimum of 20 animals by level), **AGE** is the age vector in months (included also as a quadratic effect), **u** is a vector of genetic effects, and **e** is a vector of residuals. Matrices **K**_1_, **K**_2_, and **K**_3_ are linear or Gaussian kernels for the genomic effect. We computed 3 kernels, 2 linear kernels with SNPs and HAP, and 1 Gaussian kernel with ROH. The linear kernel with SNPs was computed as K_SNP_ = **ZZ^T^**/*k*, where **Z** is a matrix of centered and standardized SNP genotypes, *k* represents the number of SNP, and **T** is the transpose matrix. The linear kernel with HAP was computed as K_HAP_ = **ZZ^T^**/*k*, where **Z** is a matrix of centered and standardized HAP and *k* represents the number of HAP. The Gaussian kernel for ROH was obtained using the average squared-Euclidean distance between ROH as follows:

$$K_{\mathrm{ROH}}=exp\left(-h\times \frac{\sum_{k=1}^{p}{\left(a_{i}-a_{j}\right)}^{2}}{\mathrm{mean}\left(\sum_{k=1}^{p}{\left(a_{i}-a_{i}\right)}^{2}\right)}\right),$$

where *a_i_* and *a_j_* are elements of 2 animals to be compared, *k* refers to a particular ROH, and *h* is the bandwidth parameter (set as 1.5) chosen over a grid of values to maximize the prediction accuracy; dividing by the mean of the squared distances ensures that the overall scale of the distances is adjusted (De los Campos et al., 2010).

Prediction models were implemented within a Bayesian framework using Markov Chain Monte Carlo methods; each model was fitted with 100,000 iterations, 30,000 burn-in and thin 5. We retained 14,000 samples for inference. All analyses were performed using the Bayesian Generalized Linear Regression package using software R 4.4.2 (BGLR, Pérez and de los Campos, 2014). To assess how well the ridge regression models could predict unseen phenotypes, a 10-fold cross-validation was conducted. Records were randomly divided into 10 nonoverlapping subsets of roughly equal size (574 records for milk yield, 550 for SCS, and 470 for days open). In each cross-validation round, 9 subsets were used as the training set to estimate fixed and random effects, whereas the remaining subset served as the testing set to evaluate the predictive performance of the models.

In addition, single-step GBLUP (**ssGBLUP**) analyses were performed for each trait using the BLUPF90 software package (Misztal et al., 2002) to estimate the variance components by REML to compare the genetic variance explained with the other methodologies. The analyses included SNPs retained after quality control, fixed effects and the animal effect as specified in model 2, and a pedigree file comprising 16,944 individuals with an average depth of at least 3 generations.

Cows were on average 24.29 ± 2.04 mo of age, produced on average 12,990 ± 2,044 kg of milk adjusted 305 d, SCS of 1.17 ± 1.02, and had on average 122.1 ± 68.4 d open. The use of genomic information yielded higher predictive performance than using only fixed effects, except for the model that included K_ROH_. There were no differences in predictive performance comparing single versus multikernel models. Kernel models fitting SNPs yielded the best predictive performance: correlations between predicted and observed phenotypes for milk yield of 0.57 (0.56–0.58, 95% CI), 0.63 for SCS (0.63–0.64, 95% CI), and 0.20 for days open (0.19–0.22, 95% CI). In the models including only fixed effects the predictive performance was 0.48 (0.47–0.49, 95% CI) for milk yield, 0.62 (0.62–0.63, 95% CI) for SCS, and 0.17 (0.16–0.19, 95% CI) for days open (Figure 1).

**Figure 1:**
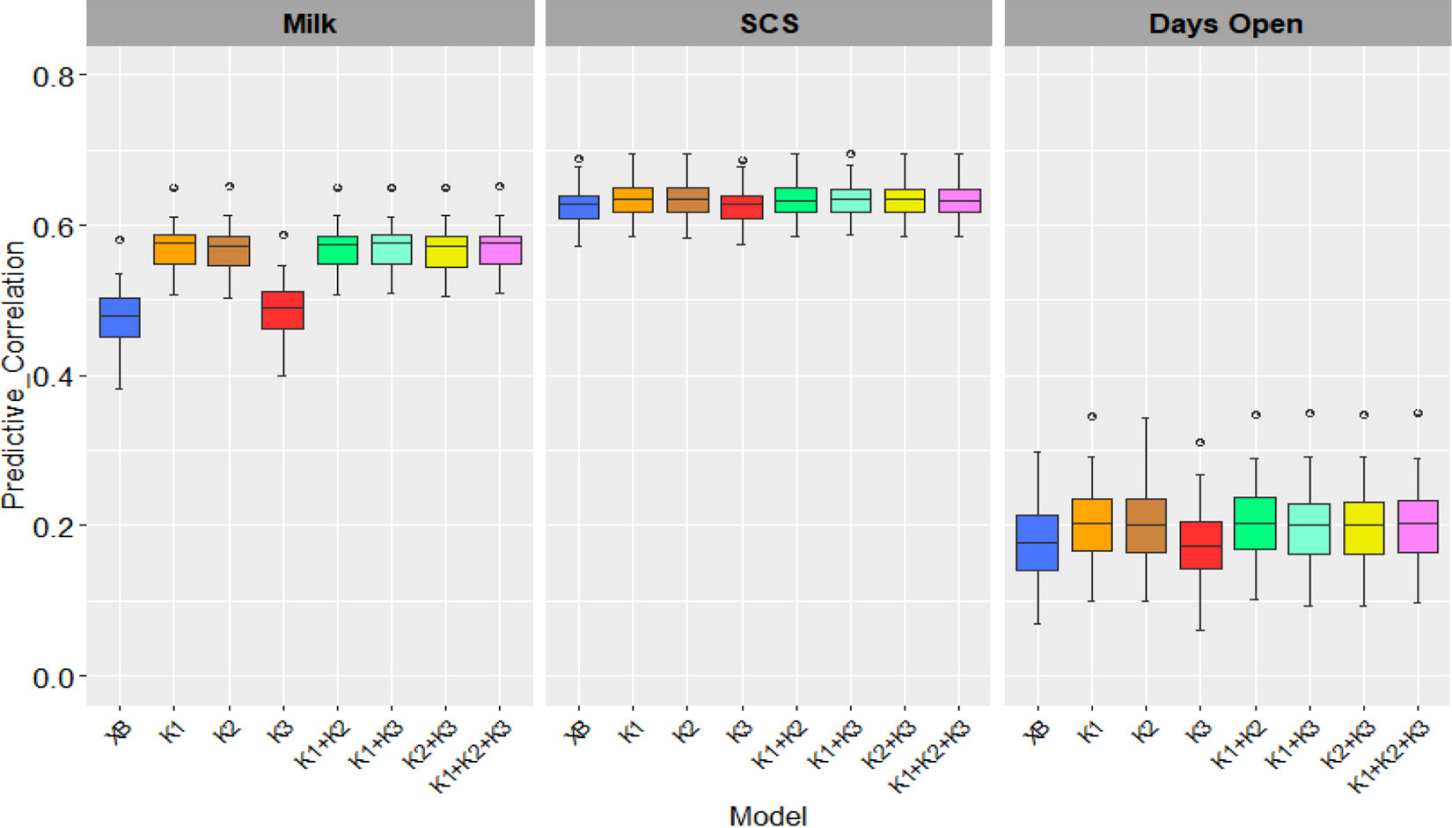
Predictive performance (Pearson correlation) in testing models for each trait. XB = model with only fixed effects; K_1_ = model with fixed effects and K_SNP_; K_2_ = model with fixed effects and K_HAP_; K_3_ = model with fixed effects and K_ROH_; K_1_ + K_2_ = model with fixed effects plus K_SNP_ and K_HAP_; K_1_ + K_3_ = model with fixed effects plus K_SNP_; and K_HAP_; K_2_ + K_3_ = model with fixed effects plus K_HAP_ and K_ROH_; K_1_ + K_2_ + K_3_ = model with fixed effects plus K_SNP_, K_HAP_, and K_ROH_. Each box represents the distribution of Pearson correlation values for the evaluated models (Q1–Q3); the horizontal line indicates the median (Q2), the whiskers represent variability, and the dots denote outliers.

The results obtained with ssGBLUP-REML of explained genetic variance indicated a higher estimation for milk yield (877,240 ± 74,775) compared with model 2, which included SNPs (861,677 ± 71,207). In contrast, for SCS and days open, the explained genetic variance was lower with ssGBLUP-REML; 0.057 ± 0.01 and 310.49 ± 74 compare with 0.07 ± 0.01 and 432.5 ± 71, respectively (Table 1). In addition, the accuracy of the prediction for milk yield was 0.73 ± 0.03, 0.55 ± 0.04 for SCS, and 0.49 ± 0.04 for days open, higher values ​​for milk production and open days compared with the results of model 2, although these are different methodologies. These findings suggest that methodologies are not trait-dependent, and the selection of a specific method should be guided by the available data and computational resources. Nonetheless, models that integrate multiple sources of genetic information tend to capture a greater proportion of genetic variance to reproductive and health traits.

**Table 1:** Total genetic variance calculated by ssGBLUP and the kernel models, and the percentage explained by each source of genomic information^1^

Trait	Model	Total $\sigma_{u}^{2}$ ± SE	% SNP	% HAP	% ROH
Milk yield	ssGBLUP	877,240 ± 74,775	100		
	K_SNP_	861,677 ± 71,207	100		
	K_HAP_	830,437 ± 70,873		100	
	K_ROH_	1,209,524 ± 225,372			100
	K_SNP_ + K_HAP_	898,422 ± 93,392	58.9	41.1	
	K_SNP_ + K_ROH_	1,226,886 ± 84,388	69.7		30.03
	K_HAP_ + K_ROH_	1,195,514 ± 86,585		68.6	31.4
	K_SNP_ + K_HAP_ + K_ROH_	1,206,846 ± 103,285	44.3	28.4	27.3
SCS	ssGBLUP	0.057 ± 0.01	100		
	K_SNP_	0.07 ± 0.01	100		
	K_HAP_	0.07 ± 0.01		100	
	K_ROH_	0.17 ± 0.04			100
	K_SNP_ + K_HAP_	0.10 ± 0.01	51.4	48.6	
	K_SNP_ + K_ROH_	0.17 ± 0.02	37.7		62.3
	K_HAP_ + K_ROH_	0.17 ± 0.02		37.0	63.0
	K_SNP_ + K_HAP_ + K_ROH_	0.17 ± 0.01	24.3	23.2	52.5
Days open	ssGBLUP	310.5 ± 74	100		
	K_SNP_	432.5 ± 71	100		
	K_HAP_	415.7 ± 67		100	
	K_ROH_	967.8 ± 303			100
	K_SNP_ + K_HAP_	545.3 ± 58	51.4	48.6	
	K_SNP_ + K_ROH_	1,035.9 ± 145	36.8		63.2
	K_HAP_ + K_ROH_	1,040.5 ± 148		35.3	64.7
	K_SNP_ + K_HAP_ + K_ROH_	1,061.4 ± 110	24.0	22.3	53.7

1 K_SNP_ = kernel with SNPs; K_HAP_ = kernel with HAP; K_ROH_ = kernel with ROH (in all the models including fixed effects). $\sigma_{u}^{2}$ = genetic variance; % SNP = percentage of the genetic variance explained by kernel with SNPs; % HAP = percentage of the genetic variance explained by kernel with HAP; % ROH = percentage of the genetic variance explained by kernel with ROH; ssGBLUP = single-step GBLUP.

Note that the variation explained (R^2^) by model 1 including only fixed effects was 0.26 ± 0.001 for milk yield, 0.41 ± 0.001 for SCS, and 0.06 ± 0.001 for days open. Interestingly, the R^2^ increased with the inclusion of 2 or more kernels, especially with the inclusion of K_SNP_ and K_ROH_: 0.68 ± 0.001 for milk yield, 0.64 ± 0.001 for SCS, and 0.50 ± 0.002 for days open, demonstrating that models that include genomic information yield a better goodness-of-fit. In addition, we found similar fixed effect estimates in each model and trait.

Although the models that included only fixed effects and the K_ROH_ for the 3 traits did not show predictive correlations at the same level as the other models that included only K_SNP_ or K_HAP_ (Figure 1), the largest estimations of genetic variance were obtained with models including K_ROH_, which is probably because we computed a Gaussian kernel. We can see in Table 1 that the inclusion of multiple kernels did not show a considerable increase in genetic variance compared with those models only including one type of kernel, specifically K_ROH_. But in the models with the combination of kernels, different percentages of genetic variance explained were found for each one; in the models with the inclusion of K_SNP_ and K_HAP_ for the 3 traits, it was K_SNP_ that showed the highest percentage of genetic variance explained (58.9%, 51.4%, and 51.4% for milk yield, SCS, and days open, respectively); for milk yield in the rest of models, K_SNP_ had the highest percentage of genetic variance explained: 44.3% in the model with 3 kernels and 69.7% in the model combined with K_ROH_. In contrast, for the health and reproductive traits, it was K_ROH_ that showed the highest percentage of genetic variance explained; for SCS in all models where the K_ROH_ was included the genetic variance was 0.17, and it was this same kernel that explained the highest percentage of genetic variance in the combined models (52.5%–53.0%). For days open, the model that included the 3 kernels was the one that showed the highest values of genetic variance ​​(1,061.4 ± 110) and the K_ROH_ was the one that had the highest percentage of genetic variance explained (53.7%).

The greater proportion of explained genetic variance observed with K_ROH_ is probably because ROH captures information on both recent and distant relationships among individuals through alleles identical by descent, without being restricted to the number of generations represented in a pedigree. Moreover, when used as markers, ROH have the advantage that multiple loci within them may be in LD with a QTL, whereas a single SNP can only reflect the LD between one locus and a QTL (Luan et al., 2014). In contrast, HAP, which tend to be inherited together because of their physical proximity, may include combinations of alleles that are not exclusively homozygous, as is the case of ROH. These alleles according to their combination (homozygous or heterozygous) could differentially influence the expression of a trait due to the LD between causal mutations and HAP alleles, or greater ability to capture short-range epistatic effects (Ferdosi et al., 2016; Hess et al., 2017).

In Table 2 we can observe that the heritability for the 3 traits was found to be similar with the models that included K_SNP_ or K_HAP_ independent or combined: 0.27 ± 0.02 to 0.29 ± 0.02 for milk yield, 0.11 ± 0.01 to 0.14 ± 0.01 for SCS, and 0.09 ± 0.01 to 0.12 ± 0.01 for days open. In contrast, in the models that included the K_ROH_ and the other kernels the heritability was 0.36 ± 0.06 to 0.39 ± 0.06 for milk yield, 0.25 ± 0.06 to 0.26 ± 0.04 for SCS, and 0.20 ± 0.06 to 0.22 ± 0.05 for days open.

**Table 2:** Heritability estimated (mean ± SE) by the kernels in genomic models and by ssGBLUP for milk yield, SCS, and days open

Item	Milk yield	SCS	Days open
Kernel^1^			
K_SNP_	0.28 ± 0.02	0.12 ± 0.01	0.10 ± 0.02
K_HAP_	0.27 ± 0.02	0.11 ± 0.01	0.09 ± 0.01
K_ROH_	0.36 ± 0.06	0.25 ± 0.06	0.20 ± 0.06
K_SNP_ + K_HAP_	0.29 ± 0.02	0.14 ± 0.01	0.12 ± 0.01
K_SNP_ + K_ROH_	0.39 ± 0.03	0.26 ± 0.04	0.22 ± 0.05
K_HAP_ + K_ROH_	0.38 ± 0.03	0.26 ± 0.04	0.22 ± 0.05
K_SNP_ + K_HAP_ + K_ROH_	0.38 ± 0.03	0.26 ± 0.04	0.22 ± 0.04
ssGBLUP	0.41 ± 0.02	0.10 ± 0.01	0.07 ± 0.01

1 K_SNP_ = kernel with SNPs; K_HAP_ = kernel with HAP; K_ROH_ = kernel with ROH (in all the models including fixed effects); ssGBLUP = single-step GBLUP.

For days open, the use of kernels shows levels of heritably higher than reported by Durán-Alvarez et al. (2023), which was 0.05 ± 0.004 in similar population of Mexican Holstein cows but without the use of genomic information and in cows with >3 lactations. Montaldo et al. (2010) reported levels of heritability of 0.01 ± 0.02 for calving interval at first lactation in Holstein cattle in Mexico, in another population of Holstein cows from the same country the heritability was of 0.06 ± 0.11 including 510 cows and with a 179 tag SNP panel (Zamorano-Algandar et al., 2021). In the case of SCS the heritability of a similar Holstein cattle population from the same country was 0.10 ± 0.02 without the inclusion of genomic information (Montaldo et al., 2010); this value was similar to the one found in the present study with the inclusion of K_SNP_ or K_HAP_, and in another Holstein cattle population from China the heritability was 0.24 ± 0.01 with the inclusion of genotypes of 984 individuals and 87,598 SNPs (Lu et al., 2023), similar to those found here with the inclusion of K_ROH_. This shows that the use of kernels contributes to increasing the estimation of the genetic variance and the heritability of the complex traits. Some studies claim that the use of Gaussian kernels can capture complex interactions of the genome, including nonadditive effects that are important for accurate phenotypic predictions compared with linear kernel that is expected to capture genetic signals through genomic relationships under additive inheritance (De los Campos et al., 2010).

Interestingly, we found that single kernel models for productive, health, and reproductive traits have good predictive abilities. That could be useful for hard-to-measure traits such as days open. To the best of our knowledge, this is the first study to apply multiple kernels for phenotypic prediction based on the construction of relationship matrices using SNPs, HAP, and ROH. The main advantage of kernel models is that they accommodate multiple sources of information provided that the kernels can be constructed from each information set (Morota and Gianola, 2014). Overall, we did not observe differences in prediction between kernels computed using SNPs and genomic-derived metrics. Remarkably, we found higher genetic variance when including Gaussian kernels for ROH, which could be explained by the mix of both additive and nonadditive effects.

## References

[bib1] Ashja A., Zorc M., Dovc P. (2024). Genome-wide association study for milk somatic cell score in Holstein Friesian cows in Slovenia. Animals (Basel).

[bib2] Baba T., Pegolo S., Mota L.F.M., Peñagaricano F., Bittante G., Cecchinato A., Morota G. (2021). Integrating genomic and infrared spectral data improves the prediction of milk protein composition in dairy cattle. Genet. Sel. Evol..

[bib3] Bhati M., Kadri N.K., Crysnanto D., Pausch H. (2020). Assessing genomic diversity and signatures of selection in original Braunvieh cattle using whole-genome sequencing data. BMC Genomics.

[bib4] Bian C., Prakapenka D., Tan C., Yang R., Zhu D., Guo X., Liu D., Cai G., Li Y., Liang Z., Wu Z., Da Y., Hu X. (2021). Haplotype genomic prediction of phenotypic values based on chromosome distance and gene boundaries using low-coverage sequencing in Duroc pigs. Genet. Sel. Evol..

[bib5] Browning B.L., Zhou Y., Browning S.R. (2018). A one-penny imputed genome from next-generation reference panels. Am. J. Hum. Genet..

[bib6] Calderón-Chagoya R., Vega-Murillo V.E., García-Ruiz A., Ríos-Utrera Á., Martínez-Velázquez G., Montaño-Bermúdez M. (2023). Discovering genomic regions associated with reproductive traits and frame score in Mexican Simmental and Simbrah cattle using individual SNP and haplotype markers. Genes (Basel).

[bib7] Chang C.C., Chow C.C., Tellier L.C., Vattikuti S., Purcell S.M., Lee J.J. (2015). Second-generation PLINK: Rising to the challenge of larger and richer datasets. Gigascience.

[bib8] Chen Z., Yao Y., Ma P., Wang Q., Pan Y. (2018). Haplotype-based genome-wide association study identifies loci and candidate genes for milk yield in Holsteins. PLoS One.

[bib9] Cortes-Hernández J.G., Ruiz-López F.J., Vásquez-Peláez C.G., García-Ruiz A. (2022). Runs of homocigosity and its association with productive traits in Mexican Holstein cattle. PLoS One.

[bib10] De los Campos G., Gianola D., Rosa G.J.M., Weigel K.A., Crossa J. (2010). Semi-parametric genomic-enabled prediction of genetic values using reproducing kernel Hilbert spaces methods. Genet. Res..

[bib11] Durán-Alvarez C., García-Ruiz A., Alonso Morales R.A., Eguiarte L.E., Ruiz-López F.D.J. (2023). Parámetros, correlaciones y tendencias genéticas de caracteres reproductivos en ganado Holstein de México. Rev. Mex. Cienc. Pecu..

[bib12] Ferdosi M.H., Henshall J., Tier B. (2016). Study of the optimum haplotype length to build genomic relationship matrices. Genet. Sel. Evol..

[bib13] García-Ruiz A., Ruiz-Lopez F.J., Wiggans G.R., Van Tassell C.P., Montaldo H.H. (2015). Effect of reference population size and available ancestor genotypes on imputation of Mexican Holstein genotypes. J. Dairy Sci..

[bib14] Hess M., Druet T., Hess A., Garrick D. (2017). Fixed-length haplotypes can improve genomic prediction accuracy in an admixed dairy cattle population. Genet. Sel. Evol..

[bib15] Karimi Z., Sargolzaei M., Robinson J.A.B., Schenkel F.S. (2018). Assessing haplotype-based models for genomic evaluation in Holstein cattle. Can. J. Anim. Sci..

[bib16] Lu X., Jiang H., Arbab A.A.I., Wang B., Liu D., Abdalla I.M., Xu T., Sun Y., Liu Z., Yang Z. (2023). Investigating genetic characteristics of Chinese Holstein cow's milk somatic cell score by genetic parameter estimation and genome-wide association. Agriculture.

[bib17] Luan T., Yu X., Dolezal M., Bagnato A., Meuwissen T.H. (2014). Genomic prediction based on runs of homozygosity. Genet. Sel. Evol..

[bib18] Martinez Boggio G., Monteiro H.F., Lima F.S., Figueiredo C.C., Bisinotto R.S., Santos J.E.P., Mion B., Schenkel F.S., Ribeiro E.S., Weigel K.A., Peñagaricano F. (2024). Host and rumen microbiome contributions to feed efficiency traits in Holstein cows. J. Dairy Sci..

[bib19] Meyermans R., Gorssen W., Buys N., Janssens S. (2020). How to study runs of homozygosity using PLINK? A guide for analyzing medium density SNP data in livestock and pet species. BMC Genomics.

[bib20] Misztal I., Tsuruta S., Strabel T., Auvray B., Druet T., Lee D.H. (2002). 7th World Congress on Genetics Applied to Livestock Production.

[bib21] Montaldo H.H., Castillo-Juárez H., Valencia-Posadas M., Cienfuegos-Rivas E.G., Ruiz-López F.J. (2010). Genetic and environmental parameters for milk production, udder health, and fertility traits in Mexican Holstein cows. J. Dairy Sci..

[bib22] Morota G., Gianola D. (2014). Kernel-based whole-genome prediction of complex traits: A review. Front. Genet..

[bib23] Mucha A., Wierzbicki H., Kamiński S., Oleński K., Hering D. (2019). High-frequency marker haplotypes in the genomic selection of dairy cattle. J. Appl. Genet..

[bib24] Pacheco H.A., Rossoni A., Cecchinato A., Peñagaricano F. (2023). Identification of runs of homozygosity associated with male fertility in Italian Brown Swiss cattle. Front. Genet..

[bib25] Pérez P., de los Campos G. (2014). Genome-wide regression and prediction with the BGLR statistical package. Genetics.

[bib26] Purcell S., Neale B., Todd-Brown K., Thomas L., Ferreira M.A.R., Bender D., Maller J., Sklar P., De Bakker P.I.W., Daly M.J., Sham P.C. (2007). PLINK: A tool set for whole-genome association and population-based linkage analyses. Am. J. Hum. Genet..

[bib27] Teissier M., Larroque H., Brito L.F., Rupp R., Schenkel F.S., Robert-Granié C. (2020). Genomic predictions based on haplotypes fitted as pseudo-SNP for milk production and udder type traits and SCS in French dairy goats. J. Dairy Sci..

[bib28] Temesgen M.Y., Assen A.A., Gizaw T.T., Minalu B.A., Mersha A.Y. (2022). Factors affecting calving to conception interval (days open) in dairy cows located at Dessie and Kombolcha towns, Ethiopia. PLoS One.

[bib29] VanRaden P.M., O'Connell J.R., Wiggans G.R., Weigel K.A. (2011). Genomic evaluations with many more genotypes. Genet. Sel. Evol..

[bib30] Zamorano-Algandar R., Sánchez-Castro M.A., Hernández-Cordero A.I., Enns R.M., Speidel S.E., Thomas M.G., Medrano J.F., Rincón G., Leyva-Corona J.C., Luna-Nevárez G., Reyna-Granados J.R., Luna-Nevárez P. (2021). Molecular marker prediction for days open and pregnancy rate in Holstein cows managed in a warm climate. Livest. Sci..

[bib31] Zayas G.A., Rodriguez E.E., Hernandez A.S., Rezende F.M., Mateescu R.G. (2024). Exploring genomic inbreeding and selection signatures in a commercial Brangus herd through functional annotation. J. Appl. Genet..

[bib32] Zhao G., Liu Y., Niu Q., Zheng X., Zhang T., Wang Z., Xu L., Zhu B., Gao X., Zhang L., Gao H., Li J., Xu L. (2021). Runs of homozygosity analysis reveals consensus homozygous regions affecting production traits in Chinese Simmental beef cattle. BMC Genomics.

[bib33] Zhu Y., Bu D., Ma L. (2022). Integration of multiplied omics, a step forward in systematic dairy research. Metabolites.

